# Optimization of differentiation and transcriptomic profile of THP-1 cells into macrophage by PMA

**DOI:** 10.1371/journal.pone.0286056

**Published:** 2023-07-17

**Authors:** Tiezhu Liu, Tao Huang, Jiajia Li, Aqian Li, Chuan Li, Xiaoxia Huang, Dexin Li, Shiwen Wang, Mifang Liang

**Affiliations:** 1 National Health Commission Key Laboratory for Medical Virology, National Institute for Viral Disease Control and Prevention, Chinese Center for Disease Control and Prevention, Beijing, China; 2 The First Affiliated Hospital of Anhui Medical University, Hefei, China; TotiCell Limited, Bangladesh, BANGLADESH

## Abstract

THP-1 monocyte, which can be differentiated into macrophages by PMA, is widely used in researches on pathogen infection and host innate immunity, but reports on the induction methods of PMA are different and lack a unified standard, and the transcriptome characteristics of macrophage compared with THP-1 cells remains unclear. In this research, we examined the differentiation effect of three factors including induction time, cell seeding density and PMA concentration by detecting the positive rate of CD14 expression. The concentration of 80ng/ml of PMA, the induction time of 24h, and the cell seeding density of 5×10^5^ cells/ml, could respectively facilitates a relatively higher CD14 positive rate in THP-1 cells. Under this optimized conditions, the CD14 positive rate of THP-1 cells can reach 66.52%. Transcriptome sequencing showed that after the above induction, the mRNA expression of 3113 genes which were closely related to cell communication, signal transduction, cell response to stimulus, signaling receptor binding and cytokine activity were up-regulated, and the top 10 genes were RGS1, SPP1, GDF15, IL-1B, HAVCR2, SGK1, EGR2, TRAC, IL-8 and EBI3. While the mRNA expression of 2772 genes which were associated with cell cycle process, DNA binding and replication and cell division, were down-regulated, and the top genes were SERPINB10, TRGC2, SERPINB2, TRGC1, MS4A3, MS4A4E, TRGJP1, MS4A6A, TRGJP2, MS4A4A. This research optimized the induction method on THP-1 cell differentiation from three aspects and delineated the transcriptomic profile of PMA-induced THP-1 cells, laying a foundation for the construction method of cell model and for the functional study of macrophage.

## Introduction

THP-1 is a human acute monocytic leukemia cell line isolated from the peripheral blood of a child with monocytic leukemia. Since its establishment in 1980 [1], THP-1 has been widely used in researches on macrophage-related signaling pathways and drug delivery [2, 3]. Compared with other leukemia cell lines such as HL-60 [4] and ML-1 [5], THP-1 has morphological and functional characteristics similar to human primary monocytes [6, 7], and has many characteristics such as easy culture and expansion in the laboratory, and stable gene background, which can facilitates the reproducibility of experimental results [8]. Therefore, THP-1 is an ideal tool for various laboratories to study pathogen infection mechanisms and host innate immunity.

Macrophages (Mφ) are innate immune cells differentiated from monocytes in the blood and play an important role in immune responses [9]. However, due to the low number of cells and the tedious isolation process, most THP-1 cells were induced to differentiate in the laboratory. CD14, a leukocyte differentiation antigen that exists on the surface of monocytes/macrophages lineage, especially in macrophages [10, 11], is a key marker of pro-inflammatory macrophage [12], and is often used as an indicator of THP-1 cell differentiation, and its expression can increase with the induction of PMA (phorbol 12-myristate 13-acetate) and other inducers [13, 14].

The most commonly used differentiation inducer for THP-1 cells is PMA, whose addition can result in cells attaching to tissue culture plastic and adopting a stellate morphology [15, 16]. These cells express some macrophage differentiation markers (CD14 and CD36) and can phagocytose and release TNF to a greater extent than untreated cells [17]. However, the current reports on PMA concentrations used in THP-1 differentiation vary from 5 ng/ml to 200 ng/ml [18–20], and reports on the induction time for THP-1 cells are also different, such as 24 h and 48 h [21–23]. At the same time, the seeding density of THP-1 cells is closely related to its differentiation effect. Therefore, it is important to determine the optimal method for efficient induction of THP-1 cells.

In this study, the induction were optimized by evaluating the differentiation effects of macrophages under three conditions which are different PMA concentrations, different induction times and different cell seeding densities, and the induction effect using the above optimized protocols was confirmed by optical microscope, flow cytometry and RNA-sequencing. Therefore, this research will establish a sound foundation for future molecular studies with THP-1 cells.

## Material and methods

### Experimental materials

THP-1 cell line was purchased from the American Type Culture Collection (ATCC, USA), and cultured at 37 °C under 5% CO_2_ in RPMI 1640 medium supplemented with 10% heat-inactivated fetal bovine serum (FBS, Life Technologies, USA) and 1% penicillin/streptomycin (Life Technologies, USA); THP-1 cells were differentiated with PMA (Sigma-Aldrich, Germany); Cell proliferation and viability were detected by CCK8 kit (Biosharp, China); CD14 antibody (Thermo Fisher, USA) was used to detect the positive rate of CD14 molecule.

### Cell proliferation activity assay

THP-1 cells were seeded in 96-well plates at a seeding density of 10^4^ cells/ml and incubated overnight at 37°C. The next day, different concentrations of PMA (0ng/ml, 25ng/ml, 50ng/ml, 100ng/ml, 200ng/ml and 300ng/ml) were added with 3 replicates for each concentration. After 24 hours, the proliferation activity of THP-1 cells was detected by CCK-8 kit. This procedure is strictly in accordance with the kit instructions to determine the toxicity range of PMA to THP-1 cells.

### PMA cell induction assay

Firstly, the cell seeding density was fixed at 1×10^5^ cells/ml, and the cells were seeded in 6-well plates. After 24 hours, THP-1 cells were stimulated with four different concentrations: 20ng/ml, 40ng/ml, 80ng/ml and 160ng/ml, and each concentration was repeated three times. Cells were harvested at 12h, 24h, and 36h after stimulation. Then flow cytometry was used to detect cells at different concentrations, and FITC-labeled anti-CD14 antibody was used to determine the optimal concentration of PMA.

Secondly, the PMA concentration was set to 80ng/ml, THP-1 cells were seeded in 6-well plates at densities of 1×10^5^ cells/ml, 5×10^5^ cells/ml, 1×10^6^ cells/ml and 5×10^6^ cells/ml, respectively. After 24 hours, 80ng/ml PMA was added to stimulate THP-1 cells. Cells were harvested 12, 24 and 36 hours after stimulation. Then cells with different seeding densities were detected by flow cytometry with FITC-labeled anti-CD14 antibody, and the cell differentiation state was observed under light microscope to determine the optimal seeding density.

Meanwhile, the optimal induction time was determined by comparing the differentiation results of 12h, 24h and 36h through the above two experiments.

### Flow cytometry

THP-1 cells were trypsinized, resuspended in PBS, centrifuged, washed 3 times with PBS, and resuspended in 100μl antibody working solution containing 1 μg FITC-labeled CD14 antibody (Invitrogen, REF: 11-0141-82) and protected from light at 4°C for 30 minutes, washed 3 times with PBS. The samples were then transferred for testing using a flow cytometer. Flow-Jo V10 software was used for data analysis.

### RT-PCR

RNA expression of genes involved in THP-1 cells differentiation were amplified and detected by RT-PCR. Whole RNA were firstly isolated at 0h, 12h, 24h and 36h after PMA induction, and the targeted RNAs were amplified using one-step RT-PCR kit(ABI, REF:AM1005). GAPDH were used as internal control.

### RNA sequencing

Total RNA was isolated from PMA induced and control cells using TRIzol reagent (Thermo Fisher, USA). RNA purity was checked using the NanoPhotometer^®^ spectrophotometer (IMPLEN, Germany) and RNA integrity was assessed using the RNA Nano 6000 Assay Kit of the Bioanalyzer 2100 system (Agilent Technologies, USA). Then RNA samples with three replicates were delivered to company (Applied protein technology, China) for RNA-sequencing on an Illumina Hiseq 2500 platform and 150 bp paired-end reads were generated.

### Bioinformatic analysis

SeqPrep (https://github.com/jstjohn/SeqPrep) was used to perform quality control for the sequences after RNA-seq. After adapters were removed, sequences with length below 25bp were discarded, followed by trimming of low quality bases and deletion of sequences with N ratios higher than 10%. The read depth, error rate (%), Q20 and Q30 values, GC-content (%) of the resulting high-quality clean reads were then evaluated.

DEGs(Differentially Expressed Genes) analysis was performed using DESeq2 package (Version 1.38.3) and edgeR package (Version 3.40.2) [24, 25], DEGs with log2FC>1 and *P*-adjust<0.05 were considered to be significantly different expressed genes, and the intersection of the two DEGs results were taken finally.

The GO (Gene Ontology) and KEGG (Kyoto Encyclopedia of Genes and Genomes) enrichment analysis of differential genes can explain the functional enrichment of differential genes and clarify the differences between samples at the gene function level. KOBAS (Version 1.32.0), Cluster Profiler R package (Version 3.4.4) and GOseq (Version 1.50.0) was used to do enrichment analysis in this research. When *P*<0.05, it is considered that the GO or KEGG function is significantly enriched [26]. KEGG is the main public database on the signaling pathway [27], and was used to conduct pathway analysis according to KEGG annotation results. Hypergeometric distribution test was used to calculate the significance of the enrichment of the genes adjacent to the differential mRNA in each pathway, and the volcano map and heat map of the differentially expressed mRNA were drawn using R package. Gene Ratio refers to the ratio of the number of differentially expressed genes to the total number of annotated genes located in the pathway. The top 20 pathway enriched were shown in this research.

### Statistical methods

Statistical analysis was performed using GraphPad Prism5 software, and *P*<0.05 was considered statistically significant. In the toxicity test of PMA on THP-1 cells, T-test was used to compare the proliferation activity of each concentration group with others and control group; Two-way ANOVA method was used to compare the corresponding induction effect of different PMA concentrations corresponding to different induction times; Two-way ANOVA was used to compare the differentiation effect of different seeding densities corresponding to different induction times.

## Results

### PMA cytotoxicity assay

**Fig 1:**
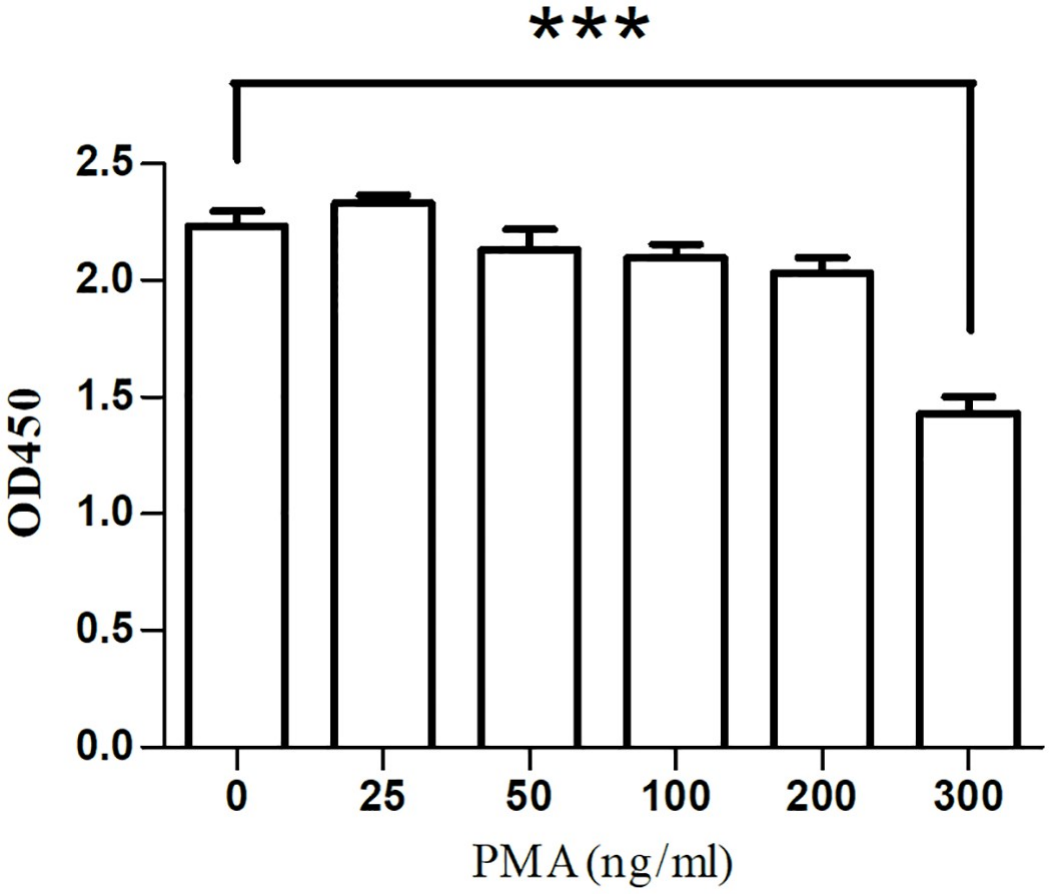
Cell proliferation activity assay in THP-1 cells for PMA. *** represents *P*<0.0001.

To investigate the cytotoxicity range of PMA, THP-1 cells were stimulated with 0-300ng/ml PMA, and cell proliferation activity was detected 24 hours later. The results showed that when the PMA concentration was lower than 200ng/ml, the cell proliferation activity had no statistical significance compared with the control group (0ng/ml). At the concentration of 300ng/ml, the proliferation activity of THP-1 cells was significantly reduced, *P*<0.0001, as shown in Fig 1. Therefore, we speculated that it is more suitable when the dosage of PMA is lower than 200ng/ml.

### Induction effect of different PMA concentrations on THP-1 cells

THP-1 cells were inoculated in 6-well cell culture plate with a seeding density of 1×10^5^ cells /ml, and cultured in incubators at 37°C, induced by PMA with concentrations of 10ng/ml, 20ng/ml, 40ng/ml, 80ng/ml, and 160ng/ml. Then cells were harvested at 12h, 24h and 36h after the addition of PMA, and then cells were detected by flow cytometry. The positive rate of CD14 was shown in Table 1. Two-way ANOVA results showed that the positive rate of CD14 was significantly different among groups with different PMA concentration (*F* = 81.59, *P*<0.0001), while the CD14 positive rate in the 80ng/ ml group (45.2%±6.9%) was higher than that in other concentration groups.

**Table 1:** Positive rates of CD14 molecules in cells with different PMA concentrations and different induction times (%, $\overset{-}{x}\pm s$).

Induction time	Concentration of PMA(ng/ml)				
	10	20	40	80	160
12h	20.0±1.3	22.7±0.8	31.6±1.3	38.4±1.0	33.1±2.5
24h	23.0±1.2	25.4±0.4	38.7±0.5	54.7±1.1	42.0±3.0
36h	20.2±2.1	24.0±1.1	37.7±0.6	42.5±1.2	34.0±1.1

Meanwhile, there was significant difference in CD14 positive rate between different induction time groups (*F* = 10.47, *P*<0.0001), and the positive rate of CD14 was higher in group with induction time of 24h (36.7%±11.7%) than other induction time groups.

### Induction effect with different seeding densities of THP-1 cells

THP-1 cells were seeded into 6-well cell culture plates at 1×10^5^ cells/ml, 5×10^5^ cells/ml, 1×10^6^ cells/ml and 5×10^6^ cells/ml and induced with PMA of 80 ng/ml. Cells were collected at 12h, 24h, and 36h, respectively, and samples with different cell seeding densities corresponding to different induction times were detected by flow cytometry. The positive rate (%) of CD14 molecule is shown in Table 2. Two-way ANOVA analysis showed that there was a significant difference in the positive rate of CD14 molecules between groups with different cell seeding densities (*F* = 48.66, *P*<0.0001), while the CD14 positive rate of 5×10^5^ cells/ml group (41.4%±8.8%) was higher than that of other seeding density groups.

**Table 2:** The positive rate of CD14 molecule in cells with different cell seeding densities and different induction times(%, $\overset{-}{x}\pm s$).

Induction time	Seeding density of THP-1 cells (cells/ml)			
	1×10^5^	5×10^5^	1×10^6^	5×10^6^
12h	29.5±3.1	35.7±2.5	25.4±0.7	33.7±1.8
24h	31.0±1.7	53.4±3.0	34.7±1.9	35.3±0.8
36h	28.4±0.4	35.4±1.8	28.0±0.7	32.7±0.8

Meanwhile, there was significant difference in CD14 positive rate between different induction time groups (*F* = 25.58, *P*<0.0001), and the positive rate of CD14 was higher in group with induction time of 24h (36.7%±11.7%) than other induction time groups.

### Induction effect under optimized method

**Fig 2:**
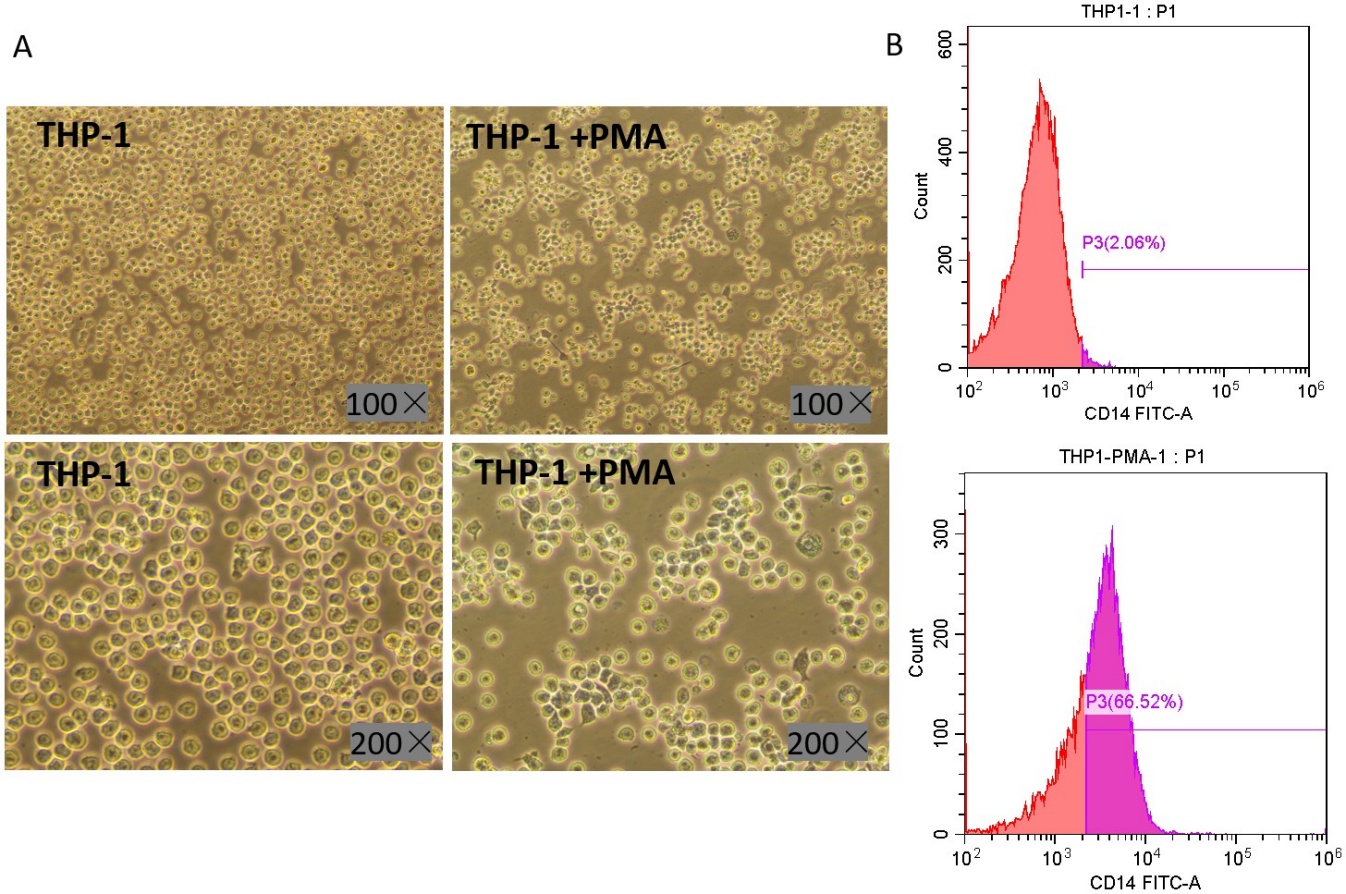
PMA-induced THP-1 differentiation effect under optimized method. **A** Morphological comparison of THP-1 cells before and after PMA induction using light microscope; **B** Comparison of CD14 positive rate of THP-1 cells before (upper panel) and after (lower panel) PMA induction detected by Flow cytometry.

According to the above optimized method, THP-1 cells were seeded into a 6-well culture plate at 5×10^5^ cells/ml, and cells were stimulated with PMA at a concentration of 80ng/ml. Cell morphology was observed under optical microscope at 24h after induction. Meanwhile, cells were collected for flow cytometry to detect CD14 positive rate. As shown in Fig 2A, THP-1 cells were in a suspended status before induction, and were characterized as small, round, and transparent. While after induction by PMA, all THP-1 cells adhered to the wall, and were characterized as larger, polygonal with extended pseudopodia, upper panel shows a 100×magnification and lower panel shows a 200× magnification. Flow cytometry showed that the positive rate of CD14 molecule was only 2.06% before PMA induction (upper panel), while it could reach 66.52% under the optimized induction conditions of this study (lower panel), as shown in Fig 2B.

### Transcriptomic comparison of THP-1 cells before and after PMA induction

After 24 h of PMA induction, total RNA was extracted from THP-1 cells and control cells, and 3 biological replicates were established for each sample. Then RNA with high quality (Table 3) were subjected to RNA sequencing. After processing the sequencing data of THP-1 cells by assembly, quality assessment, screening and filtration, more than 48 million clean reads were obtained for each group. The sequencing error rate of control group (control 1, 2, 3) was about 0.03%, and the ratio of (Q30) bases to total sequencing bases was 92.35%, 90.98%, 91.36%, respectively. For the induce 1, 2 and 3, Q30 was 93.6%, 92.6% and 91.18%, respectively, and the data quality of each group was more than 90%, indicating that the sequence results were good, as shown in Table 3.

**Table 3:** All samples of total RNA quality test results.

Sample	Raw_reads	Clean_reads	Clean_bases	Error(%)	GC (%)	Q20(%)	Q30(%)
control-1	75496620	75483598	11.21G	0.03	51.25	97.35	92.35
control-2	85797976	85793306	12.76G	0.03	51	96.89	90.98
control-3	97928512	97922980	14.57G	0.03	51.4	97	91.36
induce-1	58410812	58354742	8.65G	0.02	51.74	97.72	93.6
induce-2	48814784	48765480	7.24G	0.03	52.04	97.37	92.6
induce-3	59633056	59623838	8.87G	0.03	52.21	96.96	91.18

**Note**: Q20 and Q30 respectively represent the percentage of bases with mass value >20 or 30, and Q30 is usually taken as the evaluation standard.

**Fig 3:**
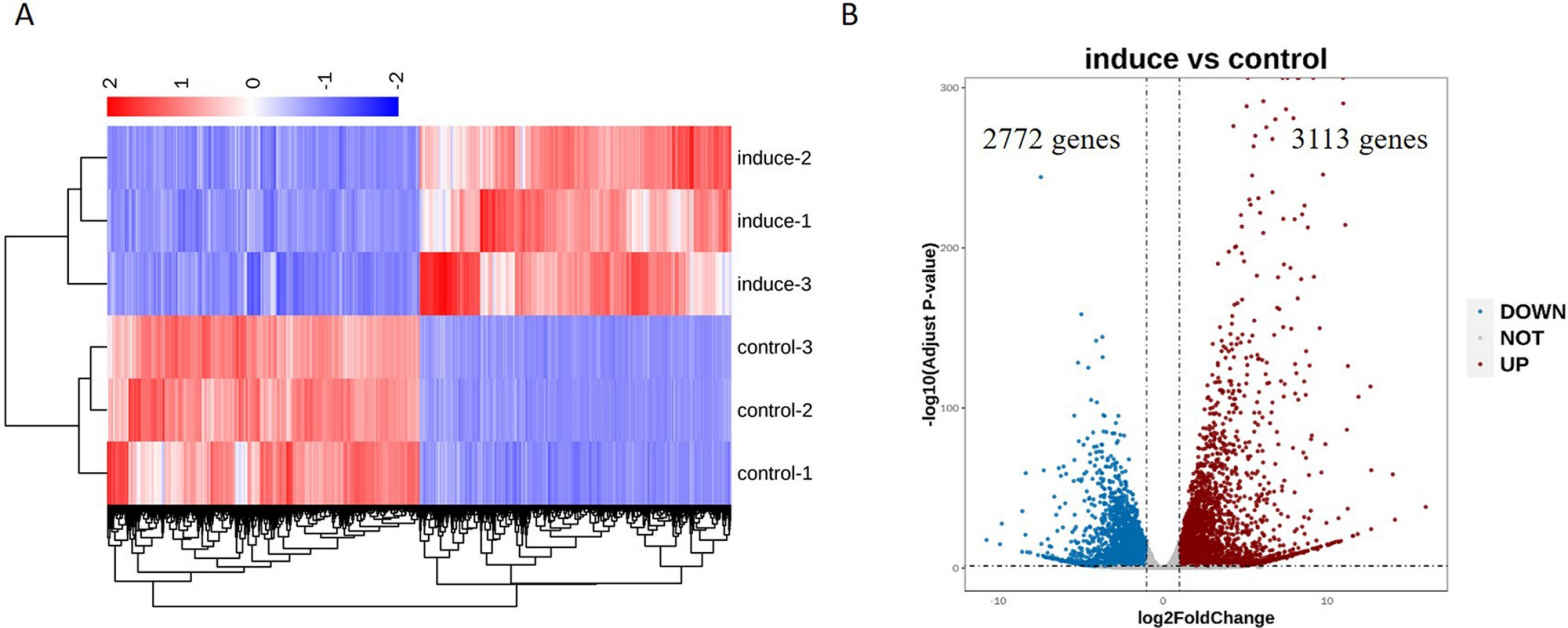
Overview of mRNAs associated with PMA induction. **A** The differential cluster analysis between different samples, DEGs were used to do the hierarchical clustering; **B** Volcano plot of up-regulation and down-regulation genes, brown circles indicate up-regulated mRNAs, and blue circles indicate down-regulated mRNAs. DEGs were used to do the hierarchical clustering.

To investigate changes in gene expression profiles induced by PMA, the Hierarchical clustering analysis was performed and the result showed that a large number of genes were robustly induced during the process of macrophage differentiation (Fig 3A). Using the criterion of *p* adjust<0.05 and log2 (fold change) >1, compared with the control samples, 3113 up-regulated mRNAs and 2772 down-regulated mRNAs were found in the induced samples, as shown in Fig 3B.

The DEGs (Different expression genes) identified in the comparison groups induce vs. control can help understand the mechanism underlying the cell response to PMA induction. The genes RGS1, SPP1, GDF15, IL-1B, HAVCR2, SGK1, EGR2, TRAC, IL-1A and EBI3 were found to be significantly up-regulated in the comparison groups induce vs. control, and the functions of these genes were found to be involved in regulation of G-protein signaling [28], stress and inflammatory response [29], regulation of macrophage activation, T-cell receptor components [30], chemokine factors and pro-inflammatory signaling cascade [31]. This up-regulated genes indicated that THP-1cells were induced to be more functional in stress, inflammatory and cell signaling response, which may provide clues for the functional study of macrophages. The top 10 up-regulated mRNAs with their functions and log2 fold change and adjusted *p* values were listed in Table 4.

**Table 4:** Top 10 up-regulated expressed mRNAs.

Gene symbol	Log2 fold change	*p* adjust	Gene function
RGS1	15.9905	3.98E-61	Attenuates the signaling activity of G-proteins
SPP1	15.1018	0	Upregulates expression of IFN-γ and IL-12
GDF15	15.0771	6.06E-291	Stress response program of cells after cellular injury
IL-1B	15.0649	0	Mediator of the inflammatory response
HAVCR2	15.0578	0	Th1-specific cell surface protein
SGK1	14.5981	0	Cellular stress response
EGR2	14.474	1.3E-221	Transcription factor
TRAC	14.0135	1.46E-218	T cell receptor alpha constant
IL-1A	13.9255	2E-213	Cytokine-cytokine receptor
EBI3	12.945	1.11E-281	Associates with IL27

It was interesting that the top 10 down-regulated mRNAs fell into 3 major families, which were serine proteinase inhibitor family (SERPINB2 and SERPINB10), MS4A family (MS4A3, MS4A4A MS4A4E, and MS4A6A) and TRG family (TRGC1, TRGC2, TRGJP1 and TRGJP2). SERPINBs usually act as potent intracellular inhibitor of GZMH (Granzyme H) by directly blocking its proteolytic activity [32, 33] and also can limit the activity of inflammatory caspases by suppressing their enzymatic activation [34]. The significant decrease of expression of SERPINB2 and SERPINB10 may provide clues that macrophage may play the same role as NK cells in the innate immune response by inducing target cell death using GZMH, and macrophage may also promote inflammatory response by activation of caspases. MS4A proteins were mainly found in monocytes/macrophages lineage, and were reported to be regulator for macrophage-dependent inflammatory responses [35]. Our result that MS4A expression decreased significantly in macrophage indicated that MS4A cluster may be negative regulators in macrophage-dependent inflammatory responses. TRG (T cell receptor gamma) family were reported to be expressed on the surface of γδ T cells, and participates in the antigen recognition [36]. In our research, the decrease of expression of TRG genes(TRGC1, TRGC2, TRGJP1 and TRGJP2) in macrophages was firstly identified, which we believe to be associated with the antigen presenting of macrophages, and the biological implications of this phenomenon remains to be studied in the future research. The top 10 down-regulated mRNAs with their functions and log2 fold change and adjusted *p* values were listed in Table 5.

**Table 5:** Top 10 down-regulated expressed mRNAs.

Gene symbol	Log2 fold change	*p* adjust	Gene function
SERPINB10	-10.736	1.63E-19	Serine proteinase inhibitor
TRGC2	-9.9184	8.47E-17	T-cell receptor-γ constant
SERPINB2	-9.8054	5.99E-30	Serine proteinase inhibitor
TRGC1	-8.5568	7.84E-38	T-cell receptor-γ constant
MS4A3	-8.3495	6.84E-62	Modulator for the G1-S cell cycle transition
MS4A4E	-8.2611	1.11E-11	Involved in cell differentiation
TRGJP1	-8.2028	8.61E-10	T-cell receptor-γ constant
MS4A6A	-8.0803	3.46 E-40	Involved in cell differentiation
TRGJP2	-8.0374	1.42E-8	T-cell receptor-γ constant
MS4A4A	-7.4286	4.91E-248	Involved in cell differentiation

**Fig 4:**
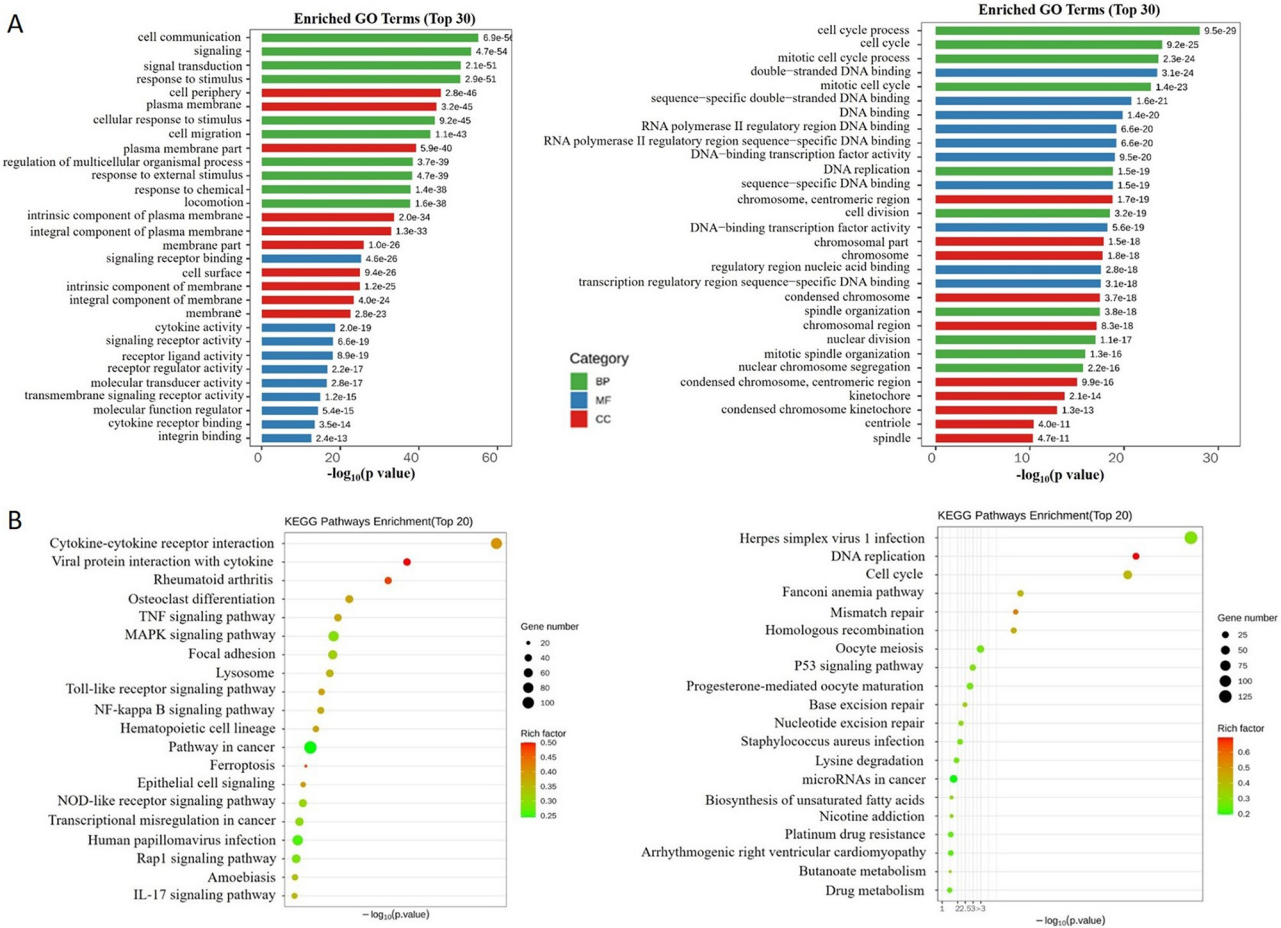
Enrichment analysis of differential expressed mRNAs in THP-1 cells after PMA induction. **A** GO function enrichment of genes in the transcriptome before and after induction, up-regulated enrichment (left panel) and down-regulated enrichment (right panel). **B** KEGG pathway enrichment of genes in the transcriptome before and after induction, up-regulated enrichment (left panel) and down-regulated enrichment (right panel).

Analysis of the DEGs (Differentially Expressed Genes) based on the biological process GO (Gene ontology) categories revealed that genes related to cellular processes and the regulation of biological processes were significantly enriched. The most significant up-regulated and down-regulated biological process terms are shown in left panel and right panel of Fig 4A, respectively. As expected, GO terms involved in response to cell communication, signal transduction, cell response to external stimulus, signaling receptor binding, cytokine activity, membrane composition changes were up-regulated following induction by PMA, while those involved in response to cell cycle process, cell proliferation, DNA binding and replication, cell division and nuclear division were down-regulated following induction by PMA. This results indicated that, after PMA induction, THP-1 cells were more sensitive to stimulatory stress and more active to intracellular signaling transduction, and cell membrane components changed in a direction that was more favorable for antigen stimulation and downstream signaling.

KEGG pathway analysis was performed to identify significantly enriched metabolic pathways or signal transduction pathways in DEGs. After PMA induction, the most significant up-regulated pathways and down-regulated pathways are shown in left panel and right panel of Fig 4B, respectively. As expected, pathways involved in cytokine and cytokine receptor interaction, viral protein interaction with cytokine, TNF signaling pathway, MAPK signaling pathway were significantly enriched, while pathways involved in DNA replication, cell cycle, mismatch repair, homologous recombination were significantly enriched. These results were consistent with the results of GO analysis.

These functional changes are closely related to the function of macrophages such as secretion of inflammatory molecules, phagocytosis of antigens and antigen presentation. Meanwhile, PMA induces mitosis and DNA replication arrest were also consistent with features of macrophage.

We also chose other key cytokines, such as IL-1β, THF-α, IL-6, IL-8, CCR5, SQSTM1, IRF3 and CD14 to perform RT-PCR detection of the RNA expression, and the results showed that only the expression of CD14, IL-1β, SQSTM1 and CCR5 increased after PMA induction (S1 Fig in S1 File), which demonstrated the expression of RNA of differentiation genes may not be the same during differentiation.

Above all, these results demonstrated that the desired induction effect of PMA to THP-1 cells was obtained, and a primary transcriptomic profile of THP-1 cells into macrophage was also established in this study.

## Discussion

Differentiation of monocytes from the common myeloid progenitor takes place in the bone marrow and is orchestrated by sequential expression and action of a specific set of transcription factors [37]. Monocytes, which leave the bone marrow and enter the circulation, are already mature cells, capable of phagocytosing microbes and secreting cytokines, but these functions need to be potentiated by further differentiation into macrophages [38].

Appropriate macrophage models are the basis for researches on the immune mechanism [39], such as screening of anti- inflammatory compounds and research on pathogen infection [40, 41]. PMA, the most commonly used inducer for THP-1 cell differentiation, can bind and activate protein kinase, further activate the transcription factor such as NF-κB [42], and promote the expression of macrophage associated proteins. A standardized induction protocol is critical for consistency and reproducibility of results between different experimenters. The previous research scheme only considered the two factors of PMA concentration and induction time, but the seeding density is also very important for the induction of cell differentiation. In this study, three elements were optimized for different PMA concentrations, different induction times, and different cell seeding densities, which will lay a sound foundation for macrophage associated researches.

Using too low a concentration of PMA will lead to insufficient differentiation, but too high a concentration of PMA will have certain toxic effects on cells. This study firstly determined that PMA concentrations lower than 200ng/ml had no effect on the proliferative activity of THP-1 cells, and then found that the concentration of 80ng/ml had a better induction effect. However, the same PMA concentration, if the cell seeding density is different during the operation, will also produce different induction effects.

Growth density is critical to cellular life, especially for cell-dependent cells [43]. Too low growth density will lead to too few growth-promoting active substances secreted by cells, which is not conducive to cell growth and differentiation [44]. Also, a big distance between cells will make it impossible to form a connection or adhesion, thus affecting cell communication. However, if the cell density is too high, there will be insufficient growth space, and the cells cannot fully adhere to the wall, and the pseudopodia cannot be prolonged or enlarged, resulting in poor differentiation effect. Therefore, it is particularly important to explore suitable growth densities for THP-1 cell differentiation. In this study, induction was performed 24 hours after inoculation, which was confirmed to produce a better differentiation in this study. It is worth noting that if the induction time points are different, the cell growth densities will also be different. Therefore, it is recommended to choose a fixed induction time point.

The length of induction time is also important for the differentiation effect of THP-1 cells. If induction is terminated prematurely, THP-1 cells will not fully differentiate. However, if the induction time was prolonged, the PMA concentration will decrease, and the adherent cells will re-round and gradually shed from the wall. In this study, after adding 80ng/ml PMA for 4h, all cells adhered to the wall, but only a few cells underwent morphological changes at this time. After addition of PMA for 12h, all THP-1 cells adhered to the wall, and the proportion of cells with morphological changes was still low. After addition of PMA for 24h, the proportion of cells with morphological changes was the largest, and then the cells gradually recovered to round shape, and finally gradually decreased.

THP-1 cells activation is associated with profound transcriptional reprogramming. Despite much progress has been made in the understanding of THP-1 cells activation, polarization, and function, the transcriptional programs regulating these processes remain poorly characterized. To confirm the induction effect of PMA with the optimized protocol in this research, RNA-sequencing was performed and revealed that cell communication, signal transduction, cell response to external stimulus, signaling receptor binding, cytokine activity, membrane composition changes were up-regulated, which indicated that compared with monocytes, macrophages have more functions in the process of innate and adaptive immunity. While those involved in cell cycle process, cell proliferation, DNA binding and replication, cell division and nuclear division were down-regulated, which indicated that macrophages have lost their ability to proliferate themselves. Meanwhile, our research that IL-1B of the top 10 up-regulated genes was also reported in the top 10 up-regulated genes in Takahide’s research [11], which indicated that IL-1B was a key gene in the differentiation of THP-1 cells or the maturation of macrophages. Our results showed that the top 10 down-regulated mRNAs fell into 3 major clusters including serine proteinase inhibitor family, MS4A family and TRG family, whose functions during monocytes/macrophage switch remains to be further studied.

Additionally, based on the transcriptome results, we also investigated the genes related to phagocytosis, TLR signaling, kinases and phosphatases, and the result showed that genes especially in pathways of TLR signaling(TLR1, TLR6, TLR7) and phagocytosis(LC3) significantly increased, demonstrating that the differentiation of THP-1 lead to increased phagocytosis and induced TLR signaling pathway.

However, there are still some improvements to be made in this study. For example, the use of CD14 molecule, as an indicator of THP-1 cell differentiation effect, may have some certain limitations. It is more comprehensive to use multiple molecules to measure the differentiation effect of THP-1 cells, but there is still a long way to go to find a standard method for the evaluation of PMA induction [45]. In addition, only 12h, 24h and 36h were selected as time points to test in this study, which need to be selected more specifically. The differentially expressed genes in transcriptome sequencing results were not exactly the same as those reported in the past [46], which may be caused by the operation bias of different experimenters or the different experimental conditions, so it will be better if samples be taken at multiple time points for RNA sequencing to make the sequencing results more convincing.

In conclusion, this study optimized the method for PMA-induced differentiation of THP-1 cells into macrophages, and provided a methodological basis for subsequent studies on the pathogenicity and innate immune mechanisms of macrophage-based viruses and other pathogens.

## Supporting information

S1 File (ZIP)
